# Editorial: Unraveling information encoding and representation in memory formation and learning

**DOI:** 10.3389/fncom.2026.1812259

**Published:** 2026-03-11

**Authors:** Fernando Montani

**Affiliations:** Instituto de Fisica La Plata (IFLP), Universidad Nacional de La Plata, CONICET CCT-La Plata (1900) La Plata, Buenos Aires, Argentina

**Keywords:** information encoding, learning, memory formation, neural networks', signal analysis

## 1 Introduction

Understanding how neural circuits encode and represent information during memory formation and learning remains one of neuroscience's most fundamental challenges. The brain's remarkable ability to transform sensory inputs into meaningful representations, store these representations over varying timescales, and retrieve them with precision requires the intricate orchestration of neural activity at multiple levels of organization. This Research Topic brings together diverse perspectives that address these mechanisms through computational, analytical, and empirical approaches.

The four contributions to this Research Topic explore critical aspects of information encoding, from the multiscale dynamics that characterize different brain states to the fundamental principles that govern how neural networks maintain stable representations while supporting continuous learning. Together, they illustrate how interdisciplinary approaches—combining information theory, computational modeling, signal processing, and neurophysiology—can illuminate the neural codes underlying memory and cognition.

## 2 Multiscale temporal dynamics across brain states

Memory consolidation critically depends on brain state transitions, particularly between wakefulness and various sleep stages. Tenti et al. employ intracranial electroencephalography (iEEG) to characterize spectral activity, neuronal avalanche dynamics, and temporal correlations across sleep-wake states. Their work reveals distinct complexity patterns associated with wakefulness, NREM sleep, and REM sleep, providing quantitative signatures of the dynamic neural activity that supports memory-related processing. By examining neural dynamics at multiple temporal scales, this study demonstrates how different frequency bands and timescales contribute to the unique information processing characteristics of each brain state. These findings have important implications for understanding how memories are consolidated during sleep and how disruptions to these state-dependent dynamics may contribute to memory disorders.

## 3 Novel computational tools for neural signal analysis

The development of sophisticated analytical methods is essential for decoding the complex patterns embedded in neural recordings. Guisande and Montani introduce the Rényi entropy-complexity causality space, a novel neurocomputational framework for detecting scale-free features in EEG and iEEG data. This approach extends traditional entropy-based analyses by incorporating statistical complexity measures and causal information flow, enabling a more nuanced characterization of neural dynamics. The authors demonstrate the tool's utility in identifying signatures of different cognitive states and pathological conditions. By providing a robust method to quantify the balance between randomness and structure in neural signals, this framework offers new avenues for investigating how information is organized in the brain and how this organization relates to memory encoding and retrieval.

## 4 Neural correlates of creative cognition in development

Creativity is a complex form of cognition that requires the flexible recombination of stored information to generate novel solutions. Krumm et al. investigate the neural basis of creativity in school-aged children through EEG power spectrum and functional connectivity analyses during creative tasks. Their findings reveal specific patterns of neural oscillations and connectivity that distinguish creative thinking from other cognitive processes. The study demonstrates enhanced alpha and theta band activity during creative tasks, alongside increased long-range functional connectivity, suggesting that creativity involves coordinated activity across distributed brain networks. This work contributes to our understanding of how developing brains encode and manipulate information in flexible ways, with implications for both educational practices and our broader understanding of how neural representations support generative cognition.

## 5 Principles of continuous learning in neural networks

A fundamental challenge in both biological and artificial neural systems is maintaining stable representations while continuously acquiring new information—the stability-plasticity dilemma. Saighi and Rozenberg address this challenge by proposing autonomous retrieval mechanisms for continuous learning in associative memory networks. Their computational model demonstrates how strategic memory retrieval can prevent catastrophic forgetting while enabling ongoing learning. The authors show that by incorporating biologically plausible retrieval dynamics, neural networks can achieve stable long-term storage of patterns while remaining adaptable to new inputs. This work bridges computational neuroscience and machine learning, offering insights into how biological memory systems balance stability and plasticity, and suggesting principles that could enhance artificial learning systems.

## 6 Synthesis and future directions

The contributions to this Research Topic collectively advance our understanding of neural information encoding through complementary lenses. They demonstrate the power of combining theoretical frameworks from information theory and signal processing innovations to reveal hidden structures in neural data, developmental perspectives to show how information processing matures, and computational models to capture fundamental principles of learning and memory.

Several themes emerge across these works. First is the importance of multiscale analysis: neural information encoding operates across multiple temporal and spatial scales, from millisecond spike patterns to hours-long consolidation processes. Second is the necessity of sophisticated analytical tools: standard linear methods often miss the nonlinear, complex dynamics that characterize real neural systems. Third is the relevance of computational modeling: abstract models help distill principles from complex biological systems and generate testable predictions.

Looking ahead, these studies point to several promising research directions. The integration of intracranial recordings with computational modeling could provide unprecedented insight into memory consolidation mechanisms. The application of novel entropy-based measures to larger datasets and different cognitive tasks could reveal general principles of neural coding. Developmental studies of information encoding could illuminate how learning systems acquire their organizational principles. Continued work on continuous learning in neural networks will be essential both for understanding biological memory and for developing more robust artificial intelligence systems.

As we continue to unravel how information is encoded and represented in neural circuits, interdisciplinary approaches such as those showcased in this Research Topic will become increasingly vital. By combining innovative analytical methods, computational models, and empirical investigations across developmental stages and brain states, we are moving closer to understanding the neural symphony that enables learning and memory.

